# Seasickness, Sea Legs, and Gravity: Suppression of Motion Sickness, Development of Sea Legs, The Role of the Striated Organelle in the Vestibular Efferent System

**DOI:** 10.3390/audiolres16030072

**Published:** 2026-05-13

**Authors:** Neil S. Longridge, Arthur I. Mallinson

**Affiliations:** Division of Otolaryngology, Department of Surgery, University of British Columbia, Vancouver, BC V6T 1Z4, Canada; nslongridge@hotmail.com

**Keywords:** sealegs, otoliths, vestibular efferents, otoconia, striated organelle

## Abstract

**Background/Objectives:** In a recent article we outlined how the vestibular efferent system connects the stereo/kinociliary complex at the apex of the macular vestibular hair cells of the inner ear and coordinates movement so that planned body movements are precisely timed to coordinate with the expected otoconial movement that the body movement induces. **Methods:** Our present article proposes an extension of this concept with details about how a sailor develops “sea legs.” The rocking motion of a boat in rough seas requires sailors to sway in order to remain vertical. This causes fluctuation in the gravity-referenced otoconial signal. **Results:** As a sailor develops sea legs, it is necessary that the routine vestibular efferent system activity (based on gravity-referenced orientation on land) is disrupted as the otoconia move with this rocking process in order to re-coordinate with the new otoconial movement. As a result, the cerebral cortex must reconfigure vestibular efferent activity so that the stereo/kinociliary complex moves in conjunction with the otoconial movement. This process is carried out via the striated organelle (STO) and is one that takes several days. Those who are unfortunate and have severe motion sickness, become extremely unwell with nausea, vomiting, severe unsteadiness, and anorexia during this time. **Conclusions:** The present article describes how “sea legs” develop and discusses why an unpleasant symptom set can accompany it. We will also outline how a new medication, a calcitonin gene-related peptide (CGRP) inhibitor, which is presently used for the treatment of vestibular dysfunction, has been shown to suppress vestibular efferent activity and may be an effective therapy for these overly symptomatic individuals.

## 1. Introduction

In a recent article published in this journal [1] we proposed a purpose and function of the vestibular efferent system, and one role of the present paper is to outline a theory about how this system is involved in developing what is colloquially referred to as “sealegs” in sailors. The inconvenience and misery caused by seasickness in the first few days at sea have long been studied by scientists. In an article by Maitland in the 1931 British Medical Journal, it was stated that the “eminently seafaring British nation” was “conspicuous for its neglect of the study of seasickness” [2]. This present article addresses how the inner ear balance system is involved with the development of “sea legs” and overcoming sea sickness. Maitland (who was a ship’s captain and also a physician) discussed the belief held at that time that seasickness was not vestibular, “as nystagmus has not been observed.” However, he also outlined that sailors who suffered from seasickness were susceptible to carsickness and train sickness and “also disliked waltzing.” He also conducted rotating chair tests and found that sailors who were seasick were bothered by this assessment, while non-susceptible sailors were not. In short, they were intolerant of any vestibular stimuli. In a follow-up discussion published at the same time, the conclusion was reached that, although the understanding was poor, “seasickness is predominantly of vestibular origin due to uneven movement” [3]. More recent research has definitively shown that all motion sickness is of vestibular, predominantly otolithic origin [4].

Most vertebrates, including humans, have five senses. These are touch, vision, hearing, smell, and taste. In the English language, intuition is referred to as the “sixth sense.” The complex inner ear balance system is the “seventh sense.” This article addresses balance function, which is vitally important, yet far less understood. This is perhaps because balance maintenance skills are acquired so early in life and the system is largely dependent on intrinsic rather than environmentally collected peripheral afferent nerve information. Our balance system is extremely complex, but it has not undergone the same level of investigation and development of knowledge as the other five senses. As it is a subtle system, it only becomes a concern when there is a malfunction which can generate acute signs and/or symptoms related to the malfunction. These include dizziness, imbalance, and associated nausea and vomiting.

## 2. Early Recognition of the Significance of Gravity

In unicellular plants, knowledge of the location of light (phototropism) is essential for the chlorophyll cycle, which results in the production of carbohydrates. In the animal kingdom, knowledge of gravity provides orientation to the top of the fluid in which an animal is floating, as light will result in a food source from the plants located there. Small calcium particles developed in unicellular animals [5], and their weight informs the animal of the location of gravity; movement upwards away from gravity is a movement toward a food source. These particles evolved over time and became embedded in the otoconial membrane of vertebrates, acting as gravity and velocity change detectors.

The inner ear vestibular system in modern mammals has two major functions. The first (and best understood) component is the semicircular canal system, which maintains ocular fixation using the vestibulo-ocular reflex (VOR) while a creature is moving. The VOR has received the most attention, and measurement of this reflex using the caloric test was the first human balance test originally developed by Barany [6]. It was helpful at lateralizing pathology [7].

## 3. Recent Methods of Assessment

Recent years have seen the development of more complex methods for measuring inner ear function. Otolithic assessment techniques have been developed for saccular assessment (cervical vestibular evoked myogenic potentials, or CVEMPs) and utricular assessment (ocular vestibular evoked myogenic potentials, or OVEMPs). In addition, head impulse tests (HIT) can be utilized to assess all three semicircular canals. All of these tests make the empirical evidence of inner ear disease much more reliable and greatly assist clinical diagnosis [8].

Research from NASA on post-flight difficulties in astronauts returning from space led to the development of Computerized Dynamic Posturography (CDP), which has also been developed as a clinical vestibular assessment [9].

Interest in the importance of the balance system related to gravity (principally the otolithic system), as outlined in [4] grew during the space race in the 1960s between Russia and the United States of America. During this period it was discovered that particularly hardy astronauts (often fighter pilots who were able to tolerate many Gs of acceleration) frequently suffered from acute nausea, often with vomiting, during their first days in space [10]. Investigations to assist with controlling this highly embarrassing, awkward, and miserably unpleasant situation in space were initially undertaken by Spoendlin and Lindeman and others [11,12]. These investigations discovered anatomical structural features of the macular systems of the inner ear.

Upon returning to Earth, the astronauts were significantly hampered by the imbalance that they experienced. This was shown to originate from the otolithic system of the ear [4] and therefore further effort was made to understand the function of the human balance organ system. It had previously been discovered by Coats [13] that when caloric testing was undertaken with the patient supine and then prone, responses were not identical in the opposite direction (as one might predict). The conclusion was that otolithic input was probably modifying nystagmus, suggesting the existence of an otolithic feedback system related to verticality and gravity.

Recent studies and scientific endeavors have increased our understanding of these structures, which function almost unnoticeably. We are learning how they orient us to gravity and what their role is in orienting us to Earth-vertical—in short, their “sensitivity to verticality,” as articulated by the authors in a recently published study [14].

Anatomical studies of the maculae of the utricle and saccule in vertebrates have been undertaken and progress has been made with regard to understanding their histology and function. Initial work outlined regional structural differences [15] and more recent work postulated a working model of the relationship between the otoconial complex and the macular sensory epithelium [16]. These investigations have been slowed and limited by the need to decalcify the temporal bone (the hardest bone in the body) in order to investigate cellular histology. This process unfortunately causes decalcification of the otoconia in the otoconial membrane of the maculae, which makes it much more difficult to understand the gravitational and 3-dimensional movement between the otoconia and the surrounding protein matrix during life. As previously mentioned, mechanical tests (CVEMPs and OVEMPs), which measured the response of the maculae of the inner ear to sound and vibration, have been developed [17,18,19]. This assessment technique resulted in an ability to assess macular function in living people.

There is an interdependent relationship between the semicircular and macular systems of the inner ear. As a result of this, foveating an object when an individual is walking can be maintained using the VOR despite the physical movement of the body and the “jerk impact” of a foot hitting the ground, causing the vibration of the whole organism. An analogy of this is the Steadicam, often used in the film industry, which “acts as a VOR” by decoupling a camera from its operator’s movement (making a visual shot look smooth and controlled) [20].

It is not entirely clear how this complex coordination is carried out. Goldberg and Fernandez [21] described the existence and anatomy of the vestibular efferent system, including observing that it is a bilateral system acting equally on both sides. Although the precise function of the vestibular efferent system is still not fully understood [22], more recent work has shown the existence of processes that inhibit at the level of the sensory receptor cell but excite at the level of the primary afferent [23]. Despite extensive research in this area, the “mysterious and perplexing action of the exceedingly complex efferent vestibular system” and the physiological significance of its function are still poorly understood. It has been shown that the system plays a role in modulating vestibular activity to carry out a specific behavioral task [23]. Perhaps this modulation helps to coordinate VOR and VSR function and minimize the difference between the two.

The striated organelle (STO) [24] is a structure that is present in primitive animals as well as mammals. It has received little attention, and its extreme importance in balance function and disorders of its function has been ignored until recently [25].

In our recent publication about falls and the activity of the vestibular efferent system [1], we outlined and described how the vestibular efferent system, using the STO, is closely coordinated with the maculae of the inner ear and body movement (as suggested in [23]) and also responds to velocity change during movement.

**Figure 1:**
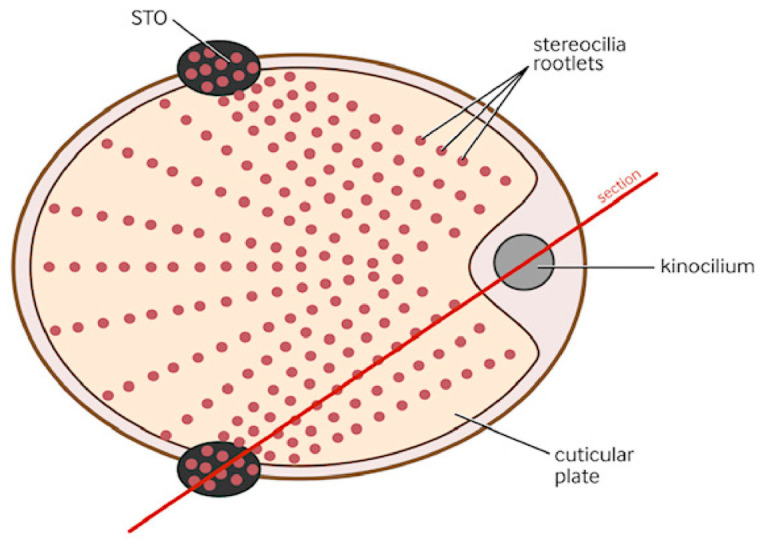
Type 1 hair cell—transverse section. (showing location of STO).

It was proposed that using the STO, the vestibular efferent system connects the distal vestibular efferent neuron to the stereo/kinociliary complex at the apex of the type 1 vestibular hair cells, situated in the maculae of the inner ear. Figure 1 is a transverse section of a type one hair cell which shows this anatomical relationship. Our previous paper detailed that the purpose of the vestibular efferent system (using the STO) is to coordinate movement so that the planned body movement is precisely timed to coordinate with the expected otoconial movement that the body movement has induced.

Our present paper expands the process colloquially referred to as developing “sea legs,” and we suggest that this process may be modulated by vestibular efferent function. When a boat is rocking on waves (or on the open ocean), a sailor must sway in the opposite direction to the boat’s motion in order to stand vertically. As a result, otoconial movement in relation to gravity fluctuates. As the sailor develops “sea legs,” it is necessary that the routine vestibular efferent system activity (based on precise gravity orientation) be disrupted to re-coordinate with the otoconial movement. During the movement of the boat, the otoconia continue their routine activity (remaining oriented to gravity). As a result of the mechanism described above, the otoconia move with this rocking process. The cerebral cortex reconfigures vestibular efferent activity so that the stereo/kinociliary complex moves with the new otoconial movement information. As one would expect, this process takes several days to complete.

We have reused the diagram from our previous paper to illustrate how this system is coordinated. We have also reused our diagrams of sectioned otoliths, outlining a static and dynamic situation.

This shows a transverse section through the cuticular plate of a type 1 vestibular hair cell. The kinocilium is on the right. The dots represent stereociliary rootlets projecting down from stereocilia, which have tip links to the kinocilium. The black areas on each side represent points of extra adherence of the STO where stereociliary rootlets tend to focus preferentially.

The red line labelled “section” in this diagram indicates a vertical section taken through the hair cell, which is illustrated in the next two diagrams.

**Figure 2:**
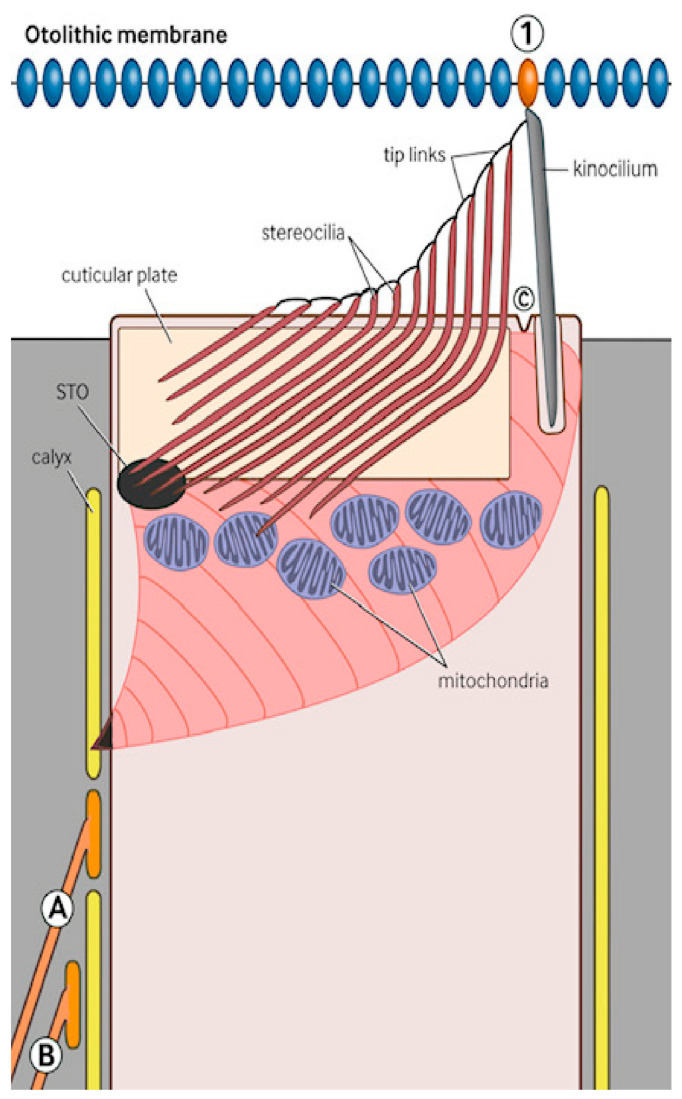
Vertical section showing a static situation.

This vertical section (Figure 2) shows the STO in pink. Neurons labeled “A” (15% of the population) synapse on the inner hair cell, and neurons labeled “B” (85% of the population) synapse on the cupula of the afferent axon. The STO extends to the cell surface at point “C” (surrounding the kinocilium). The distal calyx of the afferent vestibular axon (shown in yellow) contacts the hair cell and reaches to just below the cuticular plate. Beneath the cuticular plate of the cell is a layer of very large mitochondria (for energy supply), some of which are contacted by stereociliary rootlets that have passed through the cuticular plate. The kinocilium is attached to the otolithic membrane at site “1.” It is leaning slightly towards the center of the cell and can flex in either direction. Its contact with the rootlets means that this kinociliary/stereociliary complex can respond to a sudden, unexpected otolithic displacement.

**Figure 3:**
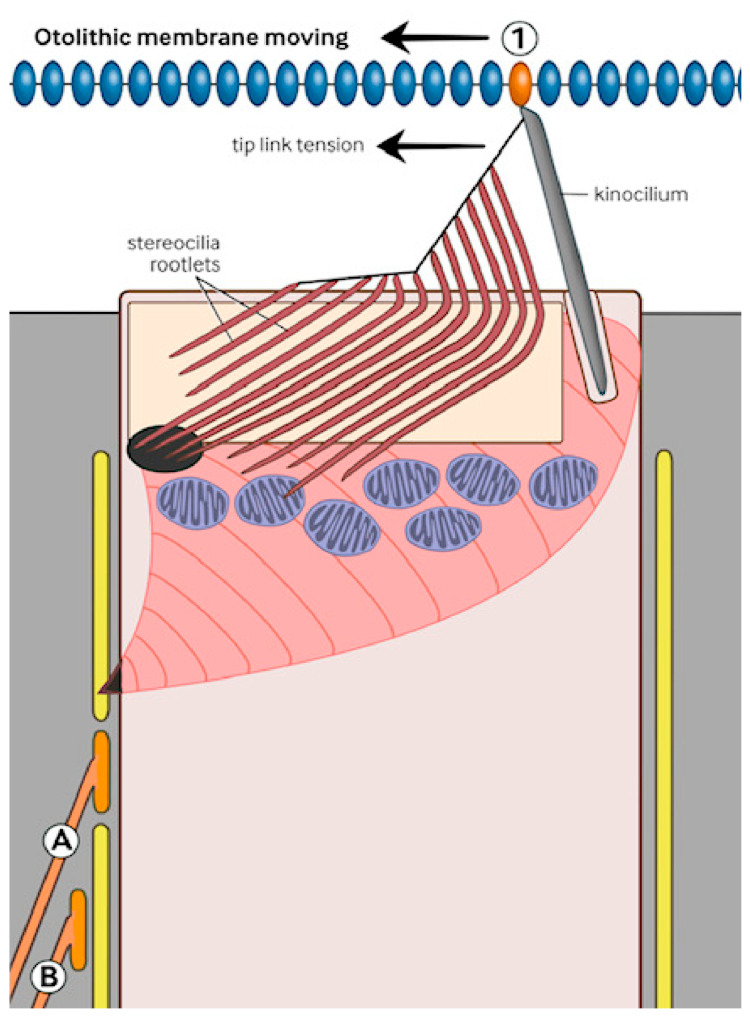
Vertical section showing a dynamic situation.

In this diagram (Figure 3) an otolithic displacement has displaced the stereocilia due to the slip. As a result, the attached kinocilium at site “1” has been displaced. This has deformed the STO and generated an appropriate response. This is maximal at right angles to the direction of the unexpected movement in the utricular striola, and the detected direction of the rapid response will allow for a protective movement preventing injury, fracture, or death. The development of “sea legs” entails the cerebral cortex reconfiguring vestibular efferent activity so that the stereo/kinociliary complex moves with the new (perhaps constant) otoconial movement information disruption, thus suppressing a conflict between the two signals.

## 4. Mechanism of Activation

The STO is located near the apex of the type 1 vestibular hair cells in zone 2 at the apex of the cupola of the primary afferent axon [24,25,26,27]. The mechanism behind this process is complex [1] and is outside the scope of the present article, which addresses sea sickness. As shown in diagram 2, the anatomy of the neurons labeled “A” and the neurons labeled “B” differs. The distal plate of the A neurons (comprising about 15% of the population) synapses directly on the inner hair cell membrane in close proximity to Zone 2 of the inner hair cell near the STO, while the B neurons (comprising 85% of the cells) synapse on the cupola of the afferent axon.

STO stimulation by the vestibular efferent system causes appropriate contraction or relaxation of the contractile protein in the STO, depending on which side of the line of polarity reversal (LPR) the hair cell is located in each macula (as shown in diagram 1). This occurs maximally at right angles to the direction of the force, resulting in movement of the stereo/kinociliary complex at the apex of the hair cells. We described how this movement is precisely coordinated in time with the body and head movement that the otoconial membrane is expecting, so that there is minimal vestibular afferent axonal firing due to this movement, unless the expected and predicted movement is incorrect [1]. This can be the result of an unpredicted external stimulus, such as:

- Bumping into an unexpected obstacle;
- Because the ground is of different firmness than expected;
- A foot slip occurring;
- A sudden, unexpected swell of the sea.

The Computerized Dynamic Posturography (CDP) balance measuring system, developed during the space race was later commercialized as a method of measuring balance function in humans [9]. It includes an estimate of how much emphasis an individual places on proprioception and vision as the main balance coordinator.

**Figure 4:**
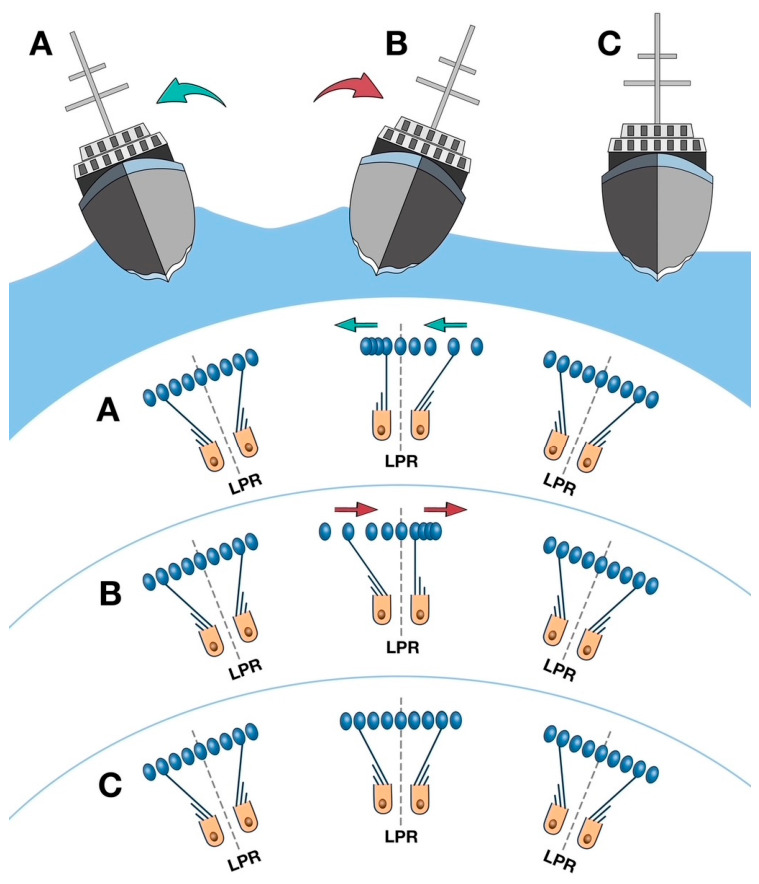
ROLLING SHIP.

This diagram (Figure 4) shows how the stereo/kinociliary complex moves as a ship rolls.

- In “A” the boat has rocked starboard (right) and the otoconia and stereo/kinociliary complex) are strung out on the side of the LPR opposite to the boat movement.
- Series “B” shows the reverse, as the boat rocks to port (left).
- Series “C” shows the lack of movement of the complex (i.e., “no stringing out”) after sea legs are acquired.

Figure 4 outlines the response of the otoconia both before and after the development of sea legs. In “A” and “B,” in the non-habituated sailor, as the boat rocks, the otoconial membrane bunches up to the side that the boat is rocking towards and is strung out to the side that the boat is rocking away from. This occurs on opposite sides of the line of polarity reversal (LPR) in each macula. This effect is maximal when roll velocity is maximal (when the boat is vertical). At the extreme ends of the roll, boat motion is zero, and the otoconia are positioned without the effect from any movement on the boat. As shown in “C,” after sea legs are established, the otoconia remain in the same position, despite the rolling motion of the boat.

When a boat is rocking or pitching, it can move in an anteroposterior (“front to back” movement), a lateral direction (“side-to-side” movement), or even in a corkscrew motion. As these movements are complex, trying to explain them in diagrammatic form is extremely difficult, so the authors have chosen to diagram a boat rolling laterally only. Sailors reading this will be familiar with the term “beam reach” and after reading this description and referring to the diagram, will be able to work out their own type of more complex ship movement (e.g., pitching, corkscrewing) and what type of stereo/kinociliary activity will occur due to vestibular efferent activity through the STO. After an individual has acquired “sea legs,” their vestibular activity is coordinated and timed with the rocking boat, so there is no difficulty with walking straight as the boat moves, in contrast to newly boarded passengers.

Visual information on a boat varies from static, non-moving images on the horizon to the situation below deck, where objects on a shelf or on a wall roll with the ship. This causes a discrepancy, and the visual imagery in some people causes the vestibular nuclei to activate the vomiting center in the brainstem (which is located anatomically close to the vestibular brainstem nuclei) with the resultant nausea and vomiting related to motion sickness.

Why does the process of acquiring sea legs take two or three days? Under normal circumstances, reliable visual information is part of the vestibular nuclear information transferred via the vestibular efferent system to coordinate routine otoconial movement. At sea, after habituation and development of sea legs, visual information no longer stimulates the vestibular efferent nuclei. In an individual who has normal balance function and tries to walk on a rolling boat prior to developing sea legs, the otoconial membranes are predicted based on a combination of vision and proprioception. This results in the individual being unstable. As described above, there is a detachment of cerebral cortical visual information from reaching the vestibular efferent system. This is because in the rolling boat situation (and subsequent inaccuracy in detecting earth vertical) these newly developed inconsistencies between visual and proprioceptive information result in a disruption of the appropriate cerebral cortical visual information reaching the vestibular efferent system. The result is the symptom set referred to as seasickness. The sufferer with severe symptoms should lie down on their side with eyes closed. This removes the visual information that is causing the conflict. They may even often fall asleep when vestibular activity is minimized during the “sea legs development” process.

As detailed in Diagram 4, in scenarios A and B, the otoconia on one side of the line of polarity reversal of the macula of the utricle in each ear are strung out, while on the other side they are bunched up. The maximum movement velocity occurs when the ship is vertical in the water during the roll. The velocity gradually decreases to zero at the maximum point of roll. The otoconia are angle-related with respect to gravity, but in someone who has developed their “sea legs,” the otoconial movement is related to proprioception and is not affected by vision during the roll. However, in someone without “sea legs,” the movement of the otoconial membrane is inconsistent across the speed of the roll, resulting in bunching up and stringing out on opposite sides of the LPR in each utricular macula.

When the balance system malfunctions in an individual at any time, they have two difficulties: one is vertigo, an inappropriate sensation of movement, and the other is imbalance, and both of these are specifically associated with nausea and even vomiting. The reason for this is that until recently *Homo sapiens* inhabited trees, and clearly a great deal of movement of tree branches rendered visual information suddenly unreliable, and the movement could result in a fall, injury, and possibly reduced reproductive success. Movement towards the center of the tree, where there are larger, less mobile branches, reduces the likelihood of this and also suppresses the unpleasant symptoms of nausea.

When a habituated sailor returns to land, he continues to have “sea legs” for up to 24 h, swaying as he walks. Even when lying in bed, there is a feeling of movement until the process reverses (once again having visual input utilized by the vestibular efferent system).

With the enhanced quality of life that normal humans now have (compared to their relatively recent ancestors), activities such as reading in a car (or even riding in a car in some susceptible individuals) can result in motion sickness [28]. It can be avoided or at least reduced in severity by looking straight ahead out the window. On board a rolling ship, the nausea occurs in a similar manner, and habituation allows this to be dampened, with the subsequent development of “sea legs.” This is a similar scenario to *Homo sapiens* in a tree, becoming more tolerant and coping better with moving branches during a storm.

There are a number of medications used to control motion sickness. A recently developed therapy for inner ear vestibular disease is calcitonin gene-related peptide (CGRP) inhibitors. CGRP is a recently recognized nervous and multisystem stimulus system with equivalency to the sympathetic and parasympathetic systems. Inhibitors of CGRP reduce dizziness of vestibular origin and assist with controlling migraine headaches [29]. These are the main uses of these medications at present. They have been shown to suppress vestibular efferent activity [30,31]. As a discrepancy between visual and proprioceptive information in the vestibular efferent system results in seasickness during the first few days at sea, the suppression of its activity has the potential to reduce the nausea and vomiting in this situation. The benefit of this is clear to someone who is extremely sensitive to motion sickness. Experiments to confirm this finding are limited. Unfortunately, the animal selected for vestibular function testing was the macaque [22], selected for its resistance to motion sickness in order to allow the experiments on the function of balance to be conducted without motion sickness interfering with the research. There is no easy animal experiment that can be conducted to look at vestibular efferent activity. The only way this could be done would be to place people known to be excessively sensitive to motion in a motion-sensitive environment (e.g., a reliably rocking boat on a consistent sea) and determine whether CGRP inhibitors reduce their sensitivity. Confirmation of this would support the theory put forward in this paper. The obvious place to undertake this is somewhere like Iceland, which has large, consistent sea swells. Much of Iceland’s population uses the sea as a livelihood (which helps to identify a motion-susceptible population and make the development of a control group simpler).

## 5. Conclusions

The vestibular efferent system is known to exist but is poorly understood. We have hypothesized that its purpose may include the development of a strategy to deal with motion sickness on a boat (colloquially described as “developing sea legs”). As a sailor does so, it is necessary for routine vestibular efferent system activity to re-coordinate with the new otoconial movements. This re-coordination of vestibular efferent activity is a process that is carried out via the STO over several days. The development of “sea legs” is often accompanied by an unpleasant set of symptoms. A CGRP inhibitor, which is presently used for the treatment of vestibular dysfunction, has been shown to suppress vestibular efferent activity and should help relieve this symptom set.

## Data Availability

The original contributions presented in this study are included in the article. Further inquiries can be directed to the corresponding author.
